# *S*-adenosyl-l-homocysteine Hydrolase: A Structural Perspective on the Enzyme with Two Rossmann-Fold Domains

**DOI:** 10.3390/biom10121682

**Published:** 2020-12-16

**Authors:** Krzysztof Brzezinski

**Affiliations:** Laboratory of Structural Microbiology, Institute of Bioorganic Chemistry, Polish Academy of Sciences, Noskowskiego 12/14, 61-704 Poznan, Poland; kbrzezinski@ibch.poznan.pl

**Keywords:** cellular methylation, protein structure, structural enzymology, protein-ligand interactions, nucleoside substrate, nucleotide cofactor

## Abstract

*S*-adenosyl-l-homocysteine hydrolase (SAHase) is a major regulator of cellular methylation reactions that occur in eukaryotic and prokaryotic organisms. SAHase activity is also a significant source of l-homocysteine and adenosine, two compounds involved in numerous vital, as well as pathological processes. Therefore, apart from cellular methylation, the enzyme may also influence other processes important for the physiology of particular organisms. Herein, presented is the structural characterization and comparison of SAHases of eukaryotic and prokaryotic origin, with an emphasis on the two principal domains of SAHase subunit based on the Rossmann motif. The first domain is involved in the binding of a substrate, e.g., *S*-adenosyl-l-homocysteine or adenosine and the second domain binds the NAD^+^ cofactor. Despite their structural similarity, the molecular interactions between an adenosine-based ligand molecule and macromolecular environment are different in each domain. As a consequence, significant differences in the conformation of d-ribofuranose rings of nucleoside and nucleotide ligands, especially those attached to adenosine moiety, are observed. On the other hand, the chemical nature of adenine ring recognition, as well as an orientation of the adenine ring around the *N*-glycosidic bond are of high similarity for the ligands bound in the substrate- and cofactor-binding domains.

## 1. Introduction

The Rossmann fold is one of the evolutionarily oldest and at the same time it is the most common protein motif responsible for selective binding of nucleosides and nucleotides [1,2,3,4,5]. Depending on the enzyme group, it can be either canonical with a central sheet formed by six parallel β chains surrounded by four α-helices, or non-canonical, e.g., containing modifications within the β sheet, altered helical environment, etc. [1]. It is present in the majority of the enzymes involved in redox reactions utilizing nicotinamide- and flavin-based cofactors, as well as in some ligases and transferases utilizing adenosine phosphates [6]. The Rossmann motif is also present in *S*-adenosyl-l-methionine (SAM)-dependent methyltransferases [7] and enzymes that bind and metabolize the by-product of the SAM-dependent methylation reaction, namely *S*-adenosyl-l-homocysteine (SAH) [8,9].

SAH strongly inhibits SAM-dependent methyltransferases and its accumulation in the cell would suppress all SAM-dependent methylation processes. Therefore, its concentration, or more precisely SAM:SAH molar ratio (as an indicator of the biological methylation activity of the cell) has to be strictly controlled [10,11,12]. In all organisms, this function is fulfilled by one or two methylation-regulating enzymes. The first one is methylthioadenosine/*S*-adenosylhomocysteine nucleosidase, with one Rossmann-fold domain subunit, hydrolyzing SAH to adenine and *S*-ribosylhomocysteine. The second enzyme is *S*-adenosyl-l-homocysteine hydrolase (SAHase), which converts SAH to adenosine (Ado) and l-homocysteine (Hcy), whose subunit contains two Rossmann-fold domains. Depending on the organism, the genome encodes both enzymes or only one of them [13].

SAHase catalyzes the reversible decomposition of SAH to Ado and Hcy, but the equilibrium of the reaction is shifted far towards SAH synthesis [14]. However, under physiological conditions, Ado and Hcy clearance is rapid and the net result is SAH hydrolysis [15]. The rapid elimination of Ado and Hcy is also important from a physiological point of view. Ado regulates numerous neurological and cardiovascular processes [16,17], whereas elevated levels of Hcy in the blood are associated with the development of various pathological conditions, including coronary thrombosis [18].

SAHases are highly conserved proteins, however, differences in polypeptide chain length are significant among them and are related to the origin of the particular enzymes. All archaeal and archaeal-type bacterial SAHase sequences are shorter (total length of approximately 400–420 amino acid residues) than those derived from most *Eukarya* and most *Bacteria* (~430–500 amino acid residues). The differences reflect the absence of eight to eleven amino acid residues at the C-terminal tail of archaeal-type SAHases and the presence of an insert of approximately forty amino acid residues in most bacterial sequences. This additional segment is also present in all plant SAHases and some other eukaryotic enzymes, but it is absent in fungal, insect, and vertebrate enzymes. The structural information on SAHases is mainly based on biocrystallographic characterization of the enzyme derived from a variety of organisms. Those studies included enzymes of eukaryotic origin from mammals (*Homo sapiens* [8], *Rattus norvegicus* [9], *Mus musculus* [19]), protozoans (*Plasmodium falciparum* [20], *Trypanosoma brucei* [PDB code 3H9U, unpublished], *Leishmania major* [3G1U, unpublished], *Cryptosporidium parvum* [5HM8 unpublished], *Acanthamoeba castellanii* [6UK3, unpublished], *Naegleria fowleri* [5V96, unpublished], and plants (*Lupinus luteus* [21]). Crystallographic studies were also conducted for some bacterial SAHases, including enzymes from *Mycobacterium tuberculosis* [22], *Bradyrhizobium elkanii* [23,24], *Cytophaga hutchinsonii* [25], *Pseudomonas aeruginosa* [26], *Burkholderia pseudomalei* (3D64, 3GLQ, unpublished), *Brucella abortus* (3N58, unpublished), and *Elizabethkingia anopheles* (6APH, unpublished). Additionally, crystal structures of an archaeal-type SAHase from hyperthermophilic bacterium *Thermotoga maritima* were determined for the enzyme in its active and inactive conformations [27,28].

**Figure 1:**
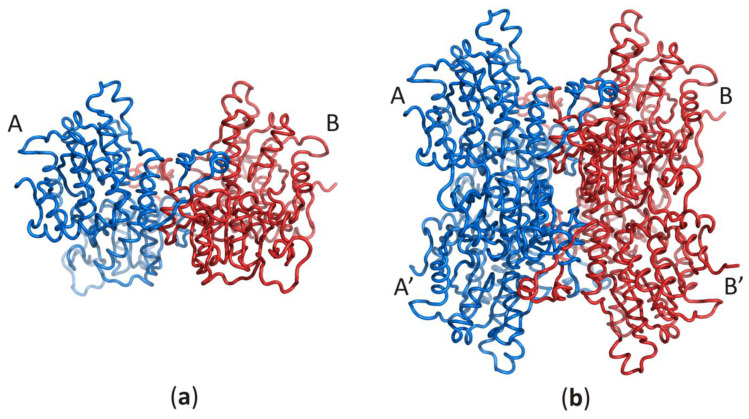
Oligomeric forms of SAHases; (**a**) The AB (or A’B’) homodimer corresponds to the active form of the plant enzyme; (**b**) The dimer of dimers (AB–A’B’) that corresponds to the active form of most SAHases of archaeal, bacterial and eukaryotic origin. The colour code indicates individual subunits: A and A’ (blue), B and B’ (red).

Despite the enzyme origin, a SAHase subunit is folded into three domains: substrate- and cofactor-binding domains, and a smaller C-terminal dimerization domain (DD). Each subunit of the active enzyme binds one nicotinamide adenine dinucleotide (NAD^+^) cofactor molecule in the cofactor-binding domain (CBD) and one substrate/product molecule (e.g., SAH/adenosine) in the substrate-binding domain (SBD). The two principal domains (substrate- and cofactor-binding) adopt the Rossmann fold and are connected by a two-part hinge element. During the catalytic cycle, the protein oscillates between two conformational states: open (when no substrate/product is bound) and closed (with substrate/product bound) [8,9]. It is of note that the oscillation frequency is directly related to the SAHase enzymatic activity and is usually strongly dependent on the presence or absence of a specific alkali metal cation coordinated at the monovalent cation binding site of the hinge region [26]. The smaller C-terminal dimerization domain extends to the adjacent domain and stabilizes the homodimer through numerous interactions with the macromolecular environment and the cofactor molecule in all SAHases of eukaryotic origin, as well as in numerous bacterial SAHases. A different situation is observed in archaeal-type SAHase from hyperthermophilic bacterium *T. maritima*, where the DD is still involved in homodimer stabilization but is too short to be involved in any interactions with the cofactor molecule bound in a neighboring subunit [27,28]. However, the homodimer is a rare active form of SAHase (Figure 1a), restricted to the plant enzyme (*Lupinus luteus*) [29,30]. The other SAHases form homotetramers or more precisely, dimers of dimers, which results from the asymmetric mode of oligomerization of the four subunits (Figure 1b).

Here, presented are the structural characteristics of the two principal domains of the SAHase subunit, which are based on the Rossmann fold. The characterization is based on the crystal structures of SAHases from both, eukaryotic and bacterial enzymes, deposited in the Protein Data Bank (PDB) [31]. Within the study, similarities and differences in the spatial arrangements of both major domains are highlighted. The study is complemented by the characterization of molecular interactions between the nucleoside (adenosine or its analogs) and the nucleotide (NAD^+^) molecules and the macromolecular environment. Additionally, a conformational analysis of the nucleoside and nucleotide molecules present in SAHase-NAD^+^-nucleoside ternary complexes has been performed.

## 2. Methods

### Databases and Software Used

Crystal structures of SAHases were acquired from PDB. The set of analyzed structural models represents SAHases from eukaryotic and prokaryotic organisms in an open or closed conformation. In most cases, four subunits of the selected enzyme are almost identical. Therefore, structural analysis and comparison studies were performed with chain A of chosen models. An exception in this respect is a subunit of SAHase from *B. elkanii*, which adopts the open conformation and corresponds to chain D of the entry 4LVC. Experimental models of SAHases selected for these studies and their brief characterization are listed in Table 1. The structural characteristic of the Rossmann motif that is present in the substrate- and cofactor-binding domains was performed based on secondary structure assignments in the PDBsum database [32]. The multiple sequence alignment was calculated in Clustal Omega [33] with amino acid sequences retrieved from the UniProt database [34]. Protein-ligand interactions were analyzed with PDBsum and COOT program [35]. Molecular figures were generated in PyMOL [36], whereas the topology diagrams were prepared with TopDraw [37]. To indicate protein-ligand interactions, amino acid residue numbers that correspond to those of plant SAHase from *Lupinus luteus* (LlSAHase) are used for detailed descriptions if not stated otherwise.

## 3. Results and Discussion

### 3.1. The Two Principal Domains of SAHase Are Based on the Rossmann Fold

Each SAHase subunit contains two principal domains that are involved in the binding of one nucleotide or nucleoside molecule. The cofactor-binding domain contains one molecule of the dinucleotide cofactor, NAD^+^, whereas the substrate-binding domain is responsible for the binding of one SAH or Ado molecule. From the evolutionary point of view, SAHases are highly conserved proteins and, unsurprisingly, the mode of nucleoside binding in SBD is almost identical among SAHases of various origins. The same is true for the cofactor binding mode in CBD.

**Figure 2:**
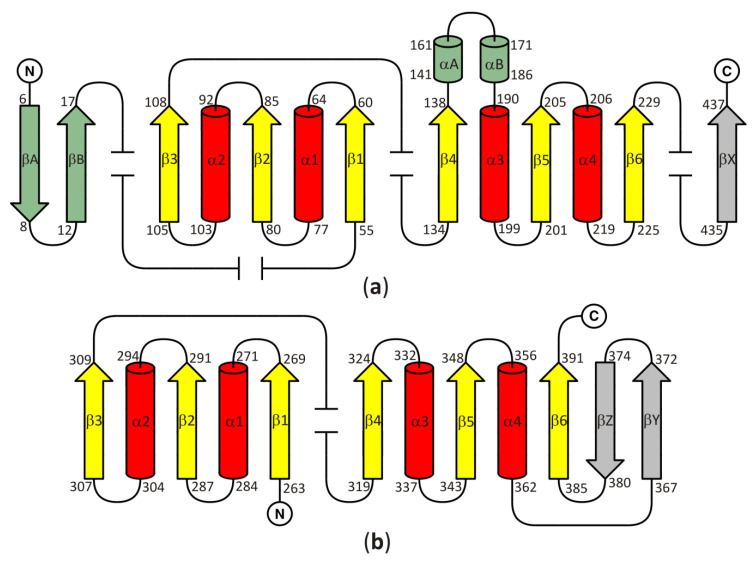
A topology diagram of the Rossmann fold in (**a**) the substrate-binding domain and (**b**) the cofactor-binding domain. Secondary structure elements present in the canonical Rossmann motif are shown in yellow and red. Additional, non-canonical β-chains present in all SAHases, in the central β-sheet, are shown in grey. The secondary structure elements present in the substrate binding-domain (SBD) of plant and numerous bacterial SAHases are shown in green. Other, variable secondary structure elements are omitted (broken lines). The numbering scheme of the secondary structure elements is based on their order in the polypeptide chain of the Rossmann fold-based domains of SAHases. For those elements that are absent in the canonical Rossmann fold, the letter designation is used. Amino acid residue numbers correspond to those of LlSAHase.

In all models of the enzyme, the SBD is based on the Rossmann fold, with a central β-sheet where the canonical six parallel β-chains (β1–β6) are arranged in the order 321456. The central β-sheet is surrounded on both sides by a pair of α helices (α1–α2 and α3–α4) and contains an additional, non-canonical parallel chain βX. Two antiparallel chains βA and βB, as well as two non-canonical, additional α helices (αA and αB) are present in SBD of plant and numerous bacterial SAHases. It should be noted that these two helices, αA and αB, correspond to the insertion region composed of approximately forty amino acid residues, present in all plant and numerous bacterial SAHases, that form a solvent-exposed region in the substrate-binding domain. CBD of SAHases is a slightly modified variant of canonical Rossmann fold. Its central β-sheet is formed by eight β chains, and six of them (β1–β6) are arranged in the order 321456 and surrounded on both sides by a pair of α helices (α1–α2 and α3–α4). Additionally, there are two short antiparallel chains βA and βB located between the canonical chains β5–β6. The topology diagram of the Rossmann fold in SBD and CBD of SAHases is shown in Figure 2a,b, respectively.

### 3.2. Ligand Binding Mode in the Substrate- and Cofactor Binding Domains

#### 3.2.1. The Role of the Loop β1, Helix α1, and Chain β2 in Ligand Binding

**Figure 3:**
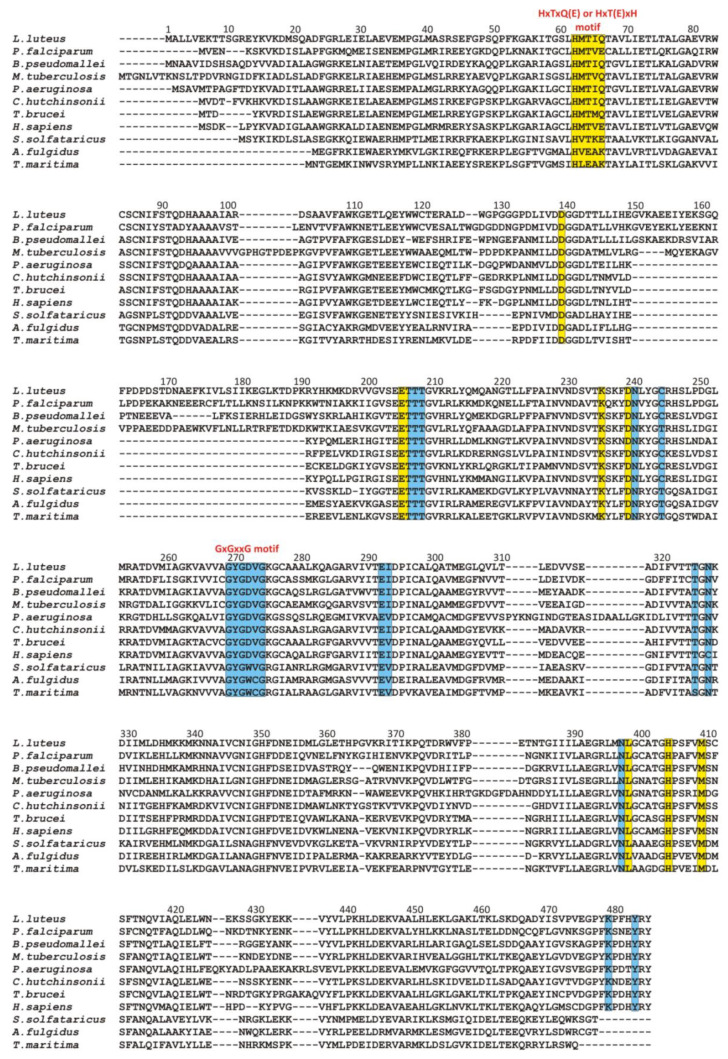
Multiple sequence alignment of selected SAHases. Residues on the yellow background are involved in a ligand binding in the substrate-binding domain, while residues on the blue background interact with the NAD^+^ cofactor molecule in the cofactor-binding domain. Two specific motifs involved in a ligand binding are highlighted. Amino acid residue numbers correspond to those of LlSAHase. The alignment includes the sequences of the following species (UniProt accession code): *Lupinus luteus* (Q9SP37), *Plasmodium falciparum* (P50250), *Burkholderia pseudomallei* (Q3JY79), *Mycobacterium tuberculosis* (P9WGV3), *Pseudomonas aeruginosa* (Q9I685), *Cytophaga hutchinsonii* (A0A6N4SNR7), *Trypanosoma brucei* (Q383X0), *Homo sapiens* (P23526), *Saccharolobus solfataricus* (P50252), *Archaeoglobus fulgidus* (O28279), and *Thermotoga maritima* (O51933).

The canonical Rossmann motif contains several regions involved in specific ligand binding. The two most characteristics are (i) the glycine-rich region corresponding to the loop β1, (located between the chain β1 and the helix α1) and the N-terminal end of the α1 helix and (ii) the acidic residue (aspartate or glutamate) that terminate the β2 chain (Figure 3). Both regions are involved in NAD^+^ binding in CBD of SAHases in a manner similar to the modes observed in various NAD^+^- or NADH-containing proteins.

**Figure 4:**
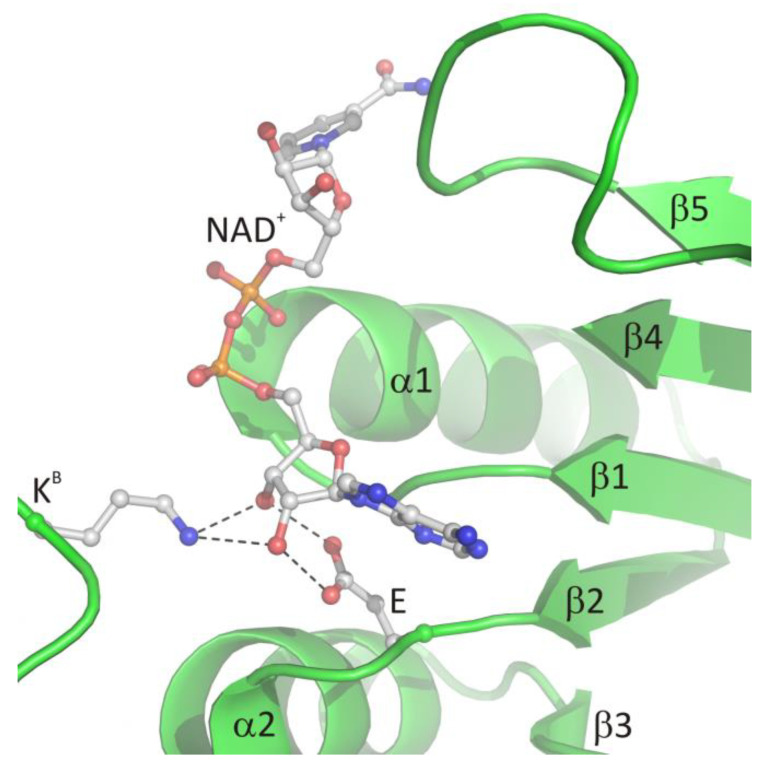
The canonical mode of NAD^+^ binding in the Rossmann fold-based CBD of SAHases. Residues located in the region of loop β1 and the N-terminal end of α1 helix, as well as the acidic residue from the β2 chain, are involved in the interactions with the cofactor molecule. Dashed lines indicate potential hydrogen bonds between 2′- and 3′-hydroxyl groups of the adenosine moiety and the conserved glutamate residue (E) from subunit A, and the conserved lysine residue from DD from the adjacent subunit B (K^B^).

The glycine-rich region contains the characteristic GxGxxG (where x may correspond to any residue) sequence signature (residues 269–274 in LlSAHase, ^Ll^G269–^Ll^G274) and interacts with the β-phosphate group of NAD^+^ molecule via electrostatic attraction between the anion (β-phosphate group) and the positive pole of the amino terminus of the α1 helix. The ε-carboxylate group of a glutamate residue in the β2 chain (^Ll^E292) is in *endo* orientation relative to the adenine ring of the cofactor and participates in the bidentate interaction with the 2′- and 3′-hydroxyl groups of the adenosine moiety of the cofactor. In consequence, the ribofuranose ring adopts, regardless of the open or closed form of the enzyme, an envelope form with C1′-*exo* conformation (pucker _1_E). A similar situation was observed for the other protein-NAD^+^ (or NADH) complexes [6]. Additionally, in all eukaryotic and numerous bacterial SAHases, a lysine residue from the DD domain of the adjacent subunit B (^Ll^K479^B^) is in the *exo* orientation relative to the adenine ring of the cofactor and participates in the formation of a bifurcated hydrogen bond with 2′- and 3′-hydroxyl groups of the ribose moiety. The canonical mode of cofactor binding in the Rossmann fold-based domain (CBD of SAHases) is shown in Figure 4.

**Figure 5:**
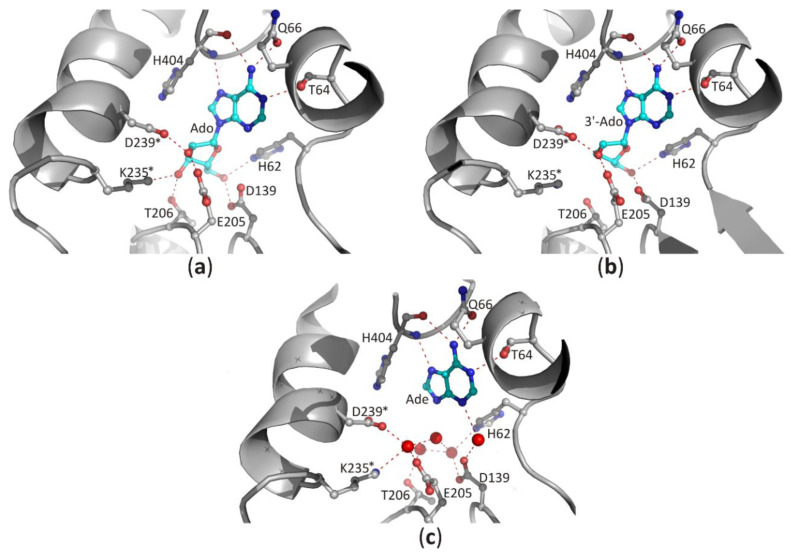
The binding mode of ligands present in the SBD domain of plant SAHase from *Lupinus luteus*: (**a**) adenosine (Ado), (**b**) 3′-deoxyadenosine (3′-dAdo), and (**c**) 2′-deoxyadenosine [26]. The potential polar interactions are marked with red dashed lines. Residues from the CBD domain are marked with stars. Water molecules are shown as red spheres.

A different situation occurs in the second domain of SAHase, which binds the nucleoside substrate (Figure 5a–c). Despite the different structures of the substrate in comparison to the cofactor molecule, the β1 loop with its immediate environment plays an important role in the binding of the nucleoside molecule (SAH or Ado). However, the molecular organization of the β1 loop, helix α1 is different (from the evolutionary point of view, this region might be poorly conserved among some proteins containing the Rossmann fold [7]). First of all, there is no glycine-rich region. Instead, eukaryotic and most bacterial enzymes contain the HxTxQ(E) sequence signature (residues 62–66 in LlSAHase; x may correspond to any residue), whereas SAHases of extremophilic origin (archaeal-type SAHases) have a different signature, namely HxT(E)xK (Figure 3). In enzymes containing the HxTxQ(E) signature, a highly conserved histidine residue (^Ll^H64 forms a hydrogen bond with a 5′-hydroxyl group of the nucleoside. It should be noted that this residue is also directly involved in the enzymatic reaction [8,21]. The other two conserved residues from this signature region are involved in the formation of hydrogen bonds with the heterocyclic nitrogen atom N1 (with the atom Oγ1 of the threonine residue, ^Ll^T64) and *exo*-amino nitrogen atom N6 (with Oε atom of the glutamine or glutamate residue, ^Ll^Q66) of the adenine moiety. In addition, the Q or E residue is involved in regulating enzyme activity by controlling the protein conformational changes that occur during the catalytic cycle [26]. So far, no experimental structure of the active form of archaeal-type SAHase-nucleoside complex has been determined. Therefore, apart from the catalytic histidine residue, the role of the other two residues from the HxT(E)xK signature remains elusive. The second distinctive element of the Rossmann fold, the acidic residue that terminates the β2 chain in CBD is absent in SBD of all SAHases. In a consequence, the region of β2 chain is not involved in any interaction with the nucleoside molecule in SBD of SAHases.

#### 3.2.2. Recognition of the Purine Ring of a Ligand and Its Orientation around *N*-glycosidic Bond

**Figure 6:**
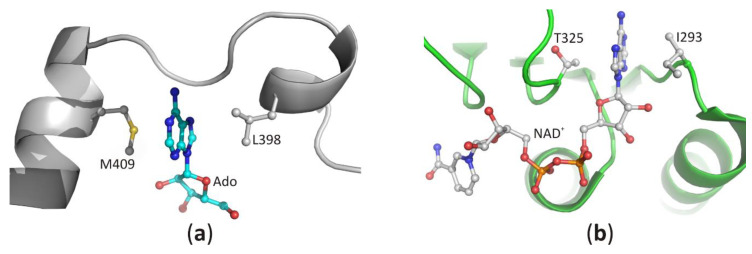
The recognition mode of the purine ring in (**a**) the substrate-binding domain and (**b**) the cofactor-binding domain of plant SAHase from *Lupinus luteus*.

Recognition of the purine ring of adenine-based ligands is very similar among the diverse proteins [39]. In SBD, both faces of the narrow binding pocket are usually formed by non-polar, sometimes also aromatic residues. The docked heterocyclic ring of the substrate is sandwiched between those residues, and its position is stabilized by C-H···π, π···π, and/or hydrophobic interactions. In the structures of SAHase-nucleoside complexes, the adenine moiety is stabilized by C-H···π and hydrophobic interactions with the side chain of the fully conserved methionine (^Ll^M409) and leucine residues (^Ll^L398). These residues are located in two adjacent α helices separated by a molecular hinge region, which is involved in protein conformational changes that occur during the catalytic cycle (Figure 6a). This indicates that a substrate-binding pocket is formed during the protein conformational change from the open to the closed-form. In CBD, the purine ring of NAD^+^ molecule is bound similarly and is sandwiched in a hydrophobic cavity formed by the side chain of valine or isoleucine residue (^Ll^I293) from the loop between the β2 chain and α1 helix and non-polar atoms of the threonine (^Ll^T325) or rarely serine (e.g., in SAHase from *T. maritima*) residue from the loop region located between chain β4 and helix α3 (Figure 6b).

**Figure 7:**
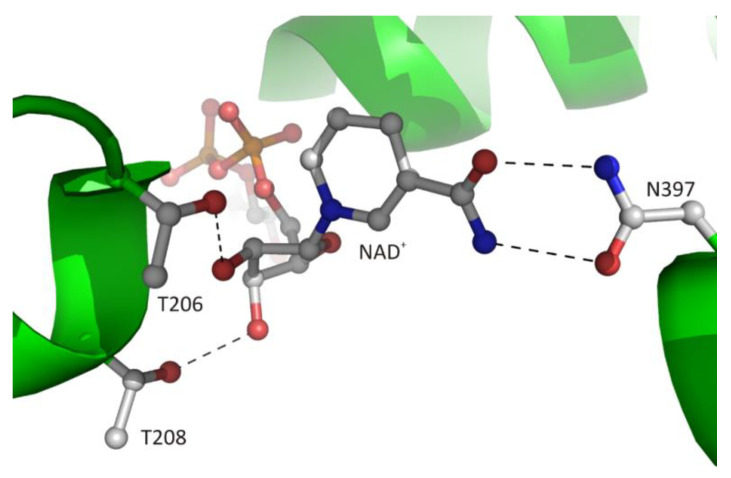
The recognition mode of the nicotinamide riboside moiety of the bound cofactor in plant SAHase from *Lupinus luteus*.

The position of the purine ring is also stabilized by polar interactions with the macromolecular environment [39]. In CBD of SAHases, the heterocyclic N7 atom of the adenine moiety of NAD^+^ forms a hydrogen bond with the Nδ2 amide nitrogen atom of the asparagine residue (^Ll^N327) located in the loop region between the chain β4 and helix α3 (Figure 7). In the case of the adenine ring of the nucleoside docked in SBD, the number of polar interactions is higher in comparison to those observed in CBD (Figure 5a–c). Herein, the purine ring is also recognized by the threonine (^Ll^T64) and glutamine (or glutamate) (^Ll^Q66) residues from the aforementioned HxTxQ(E) signature. Moreover, the main chain atoms of histidine residue (^Ll^H404) located in the molecular hinge region participate in the formation of hydrogen bonds with the *exo*-amino group (using carbonyl oxygen atom) and the heterocyclic N7 atom (using amide hydrogen atom) of purine (Figure 5a–c). The above observations show that the *exo*-amino group of adenine is a donor in two hydrogen bonds. This indicates that the adenine ring is in the preferred enamine tautomeric form [21]. In all analyzed SAHase-NAD^+^-nucleoside complexes the orientation of the purine moiety around the *N*-glycosidic bond is always *anti*, and the χ torsion angles are similar among ligands and are in a narrow range of −117.1 to −100.0° for nucleoside ligands bound in the SBD and of −111.3 to −95.1° for NAD^+^ bound in the CBD.

#### 3.2.3. Interaction of the Ligand Sugar Moiety with the Macromolecular Environment

The canonical interaction between the macromolecular environment and d-ribose moiety of the ligand is only observed in CBD and is restricted to the adenosine fragment of the cofactor, as discussed in Section 3.2.1. The second d-ribose moiety of the cofactor molecule, which is attached to nicotinamide residue, usually interacts with the side chain atoms of two conserved threonine residues located at the N-terminus of α1 helix (Figure 7). The 2′-hydroxyl group of this sugar moiety forms one hydrogen bond with the Oγ1 atom of ^Ll^T208. In the structure of archaeal-type SAHase from *T. maritima* [27], a homologous threonine residue, T140, is located in the proximity of the 2′-hydroxyl group, however, the distance between the O2′ and Oγ1 atoms is approximately 4.3 Å, so it is too large to allow a hydrogen bond formation. The second, 3′-hydroxy group of d-ribose moiety participates in the formation of one hydrogen bond with Oγ1 atom of residue ^Ll^T206. Analogous interaction is also observed in extremophilic SAHase from *T. maritima* (residue T142). In all analyzed SAHase models, the d-ribofuranose ring attached to nicotinamide residue adopts the C2′-*endo* conformation in the open, as well as the closed-form of the enzyme subunit.

In the case of the nucleoside molecule bound in the SBD, the d-ribose moiety interacts with highly conserved amino acid residues from both principal domains (SBD and CBD; residues from the CBD domain are marked with *), as shown in Figure 5a–c. The 2′-hydroxyl group forms one hydrogen bond with the ε-carboxylate group of the glutamate residue located at the end of the β5 chain, and the δ-carboxylate group of the aspartate residue from the loop region located behind the β6 chain (^Ll^E205/^Ll^D239*). The 3′-hydroxyl group interacts with the side chain of the threonine residue (^Ll^T206) located in the loop region between chain β5 helix α4, as well as with the lysine residue (^Ll^K235*) from the helical region located immediately downstream of the chain β6. In addition, the δ-carboxylate group of the aspartate residue located at the terminus of the β4 chain (^Ll^D139) and the aforementioned histidine residue (^Ll^H62) located in the β1 loop form hydrogen bonds with the 5′-hydroxyl group of the nucleoside. In the complexes of SAHases with adenosine (Figure 5a), the d-ribose ring has a strained O4′-*endo* conformation (pucker ^O^*T*_4_), which is an intermediate state between two principal modes of the furan ring (C2′-*endo* and C3′-*endo*). The strained conformation of the sugar ring is stabilized by two hydrogen bonds with the 3′-hydroxyl group of the ribose moiety. Such sugar moiety binding mode is unique among Rossmann-fold proteins. For instance, SAM-dependent methyltransferases that bind structurally similar ligands, recognize the d-ribose moiety canonically: they involve the glycine-rich region and the acidic residue from the tip of the β2 chain to bind d-ribofuranose ring that adopts the envelope form with C1-*exo* conformation [7]. On the other hand, in SAHase complexes with 3′-deoxyadenosine (3′-dAdo, Figure 5b), the sugar ring occurs in the less strained C4′-*exo* conformation (pucker _4_*T*^O^). Herein, a different conformation of the sugar ring results from the absence of a 3′-hydroxyl group, which in complexes with Ado is involved in the stabilization of the energetically unfavorable O4′-*endo* conformation through the network of hydrogen bonds with the macromolecular environment. The absence of a 3′-hydroxyl group in the ligand molecule does not significantly affect the architecture of the molecular environment of the ligand (Figure 5b). The orientation of ^Ll^K235* side chains is very similar in both complexes and is stabilized through: (i) the interaction with the macromolecular environment or (ii) interaction with the chloride anion present in the complex of SAHase from *P. aeruginosa* with 3′-dAdo, which is located near the C3′ atom of the 3′-d-deoxyribose ring. An analogous situation is observed in SAHase-adenine complexes, in which, despite the lack of the d-ribose moiety, the ligand’s macromolecular environment does not change significantly (Figure 5c). The reason for this is the presence of four water molecules (LlSAHase-adenine complex [21]) or phosphate anion (the complex of SAHase from *P. aeruginosa* with adenine [26]) located at the site usually occupied by the sugar moiety. The four oxygen atoms “mimic” the position of oxygen atoms of the d-ribose moiety and are involved in the hydrogen bond network formation in a manner analogous to that observed in SAHase complexes with Ado or 3′-dAdo. Only some small conformational differences are observed for one threonine residue (^Ll^T206) located at N-terminus of the α1 helix from CBD, depending on the ligand type.

#### 3.2.4. Interaction of Phosphate Groups of Nucleotides with the Macromolecular Environment

A characteristic feature of the canonical Rossman fold that is also observed in CBD of SAHases, is an involvement of the glycine-rich region (GxGxxG signature) in the binding of the β-phosphate group of NAD^+^ molecule. That binding is based on the interaction between the phosphate anion and the positive pole of the N-terminal end of the α1 helix. Additionally, the β-phosphate group forms a polar interaction with the hydroxyl group of the threonine residue (residue ^Ll^T207) located at the N-terminus of the α4 helix. In eukaryotic, as well as numerous bacterial SAHases, another hydrogen bond is formed with the Nδ2 atom of the asparagine residue (^Ll^N240) located in the loop region behind the β6 chain. Binding of the α-phosphate group of NAD^+^ molecule is not observed among SAHases of extremophilic origin. In other SAHases, the side chain of a tyrosine residue located in the DD domain of the adjacent subunit B (^Ll^Y483^B^) is involved in the interaction with this fragment of the cofactor.

#### 3.2.5. Interaction of the Nicotinamide Moiety with the Macromolecular Environment

The cofactor binding mode, including the nicotinamide fragment, is strictly conserved among SAHases. The nicotinamide ring is located in a narrow cavity where one face is formed by a highly conserved pair of cysteine-valine residues (^Ll^C244/^Ll^V273) or rarely by other pairs e.g., threonine-valine or threonine-cysteine in SAHase from *M. tuberculosis* or *T. maritima*, respectively. In complexes of SAHases with adenosine or its derivatives, in which the protein adopts a closed conformation, the second face of the cavity is formed by the d-ribofuranose ring of the substrate, the orientation of which allows the hydride anion to be transferred from the C3′ atom to the cofactor molecule during the catalytic reaction [8,21]. In addition, the amide group of the nicotinamide is involved in the formation of two hydrogen bonds with the Oδ1 and Nδ2 atoms of the asparagine residue (^Ll^N397) that is completely conserved among SAHases (Figure 7). This interaction ensures the stabilization of the specific orientation of the nicotinamide ring that would rotate around the *N*-glycosidic bond when the enzyme adopts a ligand-free open conformation.

#### 3.2.6. An Access to the Ligand-Binding Pockets

**Figure 8:**
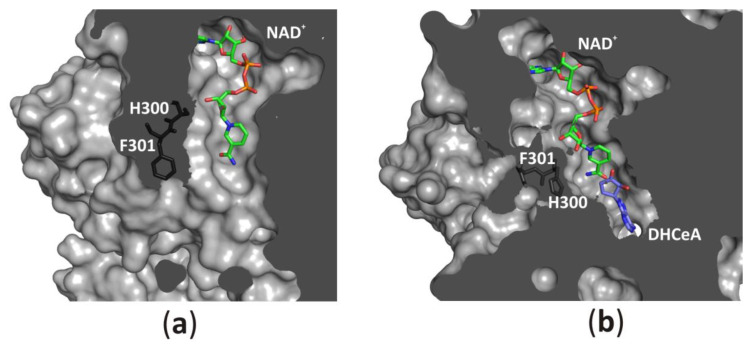
Accessibility of the active site from the solvent region is regulated by a “molecular gate” formed by a conserved His-Phe diad; (**a**) For the rat enzyme in its substrate-free state the channel is open, whereas (**b**) for the human enzyme in a complex with the adenosine analog (2′-hydroxy- 3′-ketocyclopent-4′-enyladenine, DHCeA), the channel is closed with no access to the substrate-binding site. Amino acid residue numbers correspond to those of human and rat SAHase.

The channel leading from the solvent region to the substrate-binding pocket could be opened (Figure 8a) or closed (Figure 8b) by a conserved histidine side chain and subsequent phenylalanine residue, which function as a “molecular gate”. Such access to the active site, together with protein oscillations between two conformational states, allows an unhampered substrate binding and product release during the catalytic cycle. On the other hand, the NAD^+^ molecule is tightly, but not covalently bound in the cofactor-binding pocket. All fragments of the cofactor molecule, including pyrophosphate group, nicotinamide, adenine, and d-ribose moieties are involved in polar and non-polar interactions with the macromolecular environment. It is of note, that similar modes of interactions are observed in other NAD(H)-dependent enzymes [1,6,39,40,41], however, the cofactor binding is much weaker in those enzymes in comparison to SAHases. A closer inspection of numerous NAD(H)-containing Rossmann-fold enzymes indicates that in SAHases, access of the cofactor molecule to the solvent region is significantly limited (Figure 8a,b). In the open state of SAHase, the pyrophosphate group and nicotinamide moiety could interact with water molecules from the solvent area, whereas in the closed, substrate-bound form of the enzyme only the pyrophosphate group could be involved in such interactions. Other fragments of the NAD^+^ molecule are tightly surrounded by neighboring amino acid residues, preventing the detrimental release of the cofactor during the enzymatic reaction. It is of note, that an influx of a fresh cofactor portion from an environment is not necessary, as the reactive, oxidized state of the cofactor, NAD^+^, is regenerated during the catalytic cycle [8,9,21,42].

## 4. Conclusions

So far, around fifty crystal structures of SAHase-NAD^+^-nucleoside ternary complexes were determined and are available in the PDB. Moreover, binding modes of particular ligands present in SBD and CBD were described in detail for the enzymes of various origins. However, none of those studies were to compare: (i) two major domains of SAHase subunit, (ii) recognition modes of nucleoside and nucleotide ligands bound in a SAHase subunit, and (iii) conformation of ligand molecules present in SBD and CBD of the subunit of SAHase that derived from various organisms, to highlight any possible similarities and/or differences. It is of note that comparative studies of SAHases with other Rossmann-fold NAD(H)-dependent enzymes, until now, were also elusive. Therefore, to fill this gap, detailed structural and comparative characterization of two major SAHase domains from a variety of eukaryotic and prokaryotic organisms has been performed within this review. This study indicates, that even though two principal, Rossmann-fold domains of the SAHase subunit (SBD and CBD) are structurally similar to each other, the binding modes of a nucleoside substrate in SBD and the nucleotide NAD^+^ cofactor in CBD differ significantly. The canonical protein-adenosine-based ligand interaction, similar to that observed in other nucleoside/nucleotide-binding Rossmann-fold proteins is observed only in the CBD domain. On the other hand, the recognition of the adenine ring of the ligand is similar in geometry and chemical nature in SBD and CBD and does not differ significantly from that observed in other Rossmann-fold enzymes. Moreover, the different macromolecular environment of the binding cavity in SBD and CBD does not significantly affect the orientation of the adenine ring around the *N*-glycosidic bond. Herein, the *anti*-orientation is observed for both, nucleoside and cofactor ligands. On the other hand, significant differences in the conformation of the d-ribofuranose ring are observed. d-Ribofuranose ring attached to adenine adopts O4′-*endo* or C1′-*exo* conformation in Ado or NAD^+^ molecule, respectively. The sugar ring attached to nicotinamide residue adopts C2′-*endo*.

A detailed structural comparison of numerous members of NAD(H)-dependent enzymes indicates that the sugar moiety of the substrate molecule present in the SBD of SAHases is recognized in a unique, non-canonical manner. On the other hand, the canonical binding mode is observed for NAD^+^ molecule present in the CBD of SAHases. Numerous polar and non-polar interactions between the NAD^+^ molecule and macromolecular environment are similar in SAHases and other NAD(H)-containing proteins. However, the cofactor binding is much stronger in SAHases in comparison to other enzymes. In the CBD of SAHases, the NAD^+^ molecule is very tightly surrounded by neighboring amino acid residues and, as a consequence, its release from the enzyme is significantly limited.

The above observations raise an intriguing question: how SAHase, the protein with two Rossmann-folds, had evolved during the development of life on Earth?

## Figures and Tables

**Table 1:** Selected models of SAHases used in this study and their basic characterization, including a type of nucleoside bound in the substrate-binding domain of the enzyme, subunit conformation, and the code to Protein Databank (PDB) coordinates.

Organism	Nucleoside Ligand Bound	Conformation	PDB Entry
*Homo sapiens*	neplanocin	closed	1LI4 [38]
*Rattus norvegicus*	none	open	1B3R [9]
*Mus musculus*	Ado	closed	5AXA [19]
*Plasmodium falciparum*	Ado	closed	1V8B [20]
*Trypanosoma brucei*	adenine	closed	3H9U
*Leishmania major*	Ado	closed	3G1U
*Cryptosporidium parvum*	Ado	closed	5HM8
*Acanthamoeba castellanii*	Ado	closed	6UK3
*Naegleria fowleri*	Ado	closed	5V96
*Lupinus luteus*	Ado	closed	3OND [21]
*Lupinus luteus*	adenine	closed	3ONE [21]
*Lupinus luteus*	3′-deoxyadenosine	closed	3ONF [21]
*Mycobacterium tuberculosis*	Ado	closed	3CE6 [22]
*Bradyrhizobium elkanii*	Ado	Closed ^1^	4LVC [23]
*Bradyrhizobium elkanii*	none	Open ^1^	4LVC [23]
*Cytophaga hutchinsonii*	Ado	closed	6GBN [25]
*Pseudomonas aeruginosa*	Ado	closed	6F3M [26]
*Pseudomonas aeruginosa*	3′-deoxyadenosine	closed	6F3P [26]
*Pseudomonas aeruginosa*	adenine	closed	6F3Q [26]
*Burkholderia pseudomalei*	none	open	3D64
*Burkholderia pseudomalei*	9-β-d-arabinofuranosyl-adenine	closed	3GLQ
*Brucella abortus*	Ado	closed	3N58
*Elizabethkingia anopheles*	Ado	closed	6APH
*Thermotoga maritima*	none	open	3X2E [27]
*Thermotoga maritima*	none	Closed ^2^	3X2F [27]

^1^ For the 4LVC model three subunits of the tetrameric enzyme bind Ado molecule and adopt the closed conformation, whereas, one subunit does not bind a ligand an adopt the open conformation. ^2^ Despite the fact that nucleoside molecule is absent in the substrate-binding domain, four subunits adopt the closed conformation.
